# Supplementary material for: WordCommentsAnalyzer: A windows software tool for qualitative research
Source: F1000Res. 2018 Sep 4;7:536. Originally published 2018 May 3. [Version 2] doi: 10.12688/f1000research.14819.2 (PMC6137411; doi:10.12688/f1000research.14819.2)
Supplement: Supplementary file 3 [file f1000research-7-17614-s0002.tgz › b2a79496-4ada-47c4-9682-a99ce867836d.zip!/Eight Iranian Health Journals/Journal of Health System Research _35.docx]

© Journal of Health System Research . This work is licensed under CC BY-NC 4.0 (https://creativecommons.org/licenses/by-nc/4.0/).

Volume & Issue: دوره 8، شماره 4؛ 1391

Title: بررسی عوامل مؤثر بر مصرف ماهی بر اساس سازه‌های مدل‌های آموزش سلامت

http://hsr.mui.ac.ir/index.php/jhsr/article/view/341

بیماری‌های قلبی- عروقی به عنوان اولین عامل مرگ و میر در ایران شناخته شده است و تغذیه به عنوان اولین و مهم‌ترین عامل در بروز این بیماری‌ها معرفی شده است؛ به طوری که از مجموع 800 مورد مرگ و مير روزانه در كشور، 300 مورد مستقيم به تغذيه نامناسب مربوط مي‌شود. در این میان، ماهی به عنوان غذای سلامتی مطرح است و مقدار مصرف آن در يك رژيم غذايي متعادل، دوبار در هفته توصيه شده است. متأسفانه مصرف سرانه ماهي در ايران بين 9-5/7 كيلوگرم در سال برآورد شده است و در اغلب استان‌ها این میزان به کم‌تر از 4 كيلوگرم می‌رسد. شناسایی عوامل مؤثر بر مصرف ماهی اولین گام در اصلاح و بهبود این رفتار است. تحقیق‌ها نقش عواملی مانند بو و مزه ماهی، ترس از استخوان‌های ریز ماهی، توانایی در آماده‌سازی و پخت و فواید درک‌شده را ثابت کردند. تأثیر نگرش فرد در مصرف ماهی غیر قابل انکار است. مدل‌های رایج در آموزش سلامت مؤلفه‌های اساسی را شناسایی نموند که در ایجاد و یا تغییر یک رفتار مؤثرند و به ضریب وزنی هر یک از این مؤلفه‌ها و سازه‌ها در جوامع مختلف تأکید نمودند. کالبدشناسی علل رفتار از هنرهای مدل‌های آموزش بهداشت می‌باشد. الگوها و مدل‌هایی که فرد را مسؤول رفتار می‌داند، شامل تئوری رفتار برنامه‌ریزی شده، الگوی اعتقاد بهداشتی، عمل منطقی و مراحل تغییر می‌باشد. این بررسی درصدد تبیین عوامل مؤثر بر مصرف ماهی بر اساس مدل‌ها و الگوهای رایج در آموزش بهداشت است.

Title: تأثیر مکالمه با تلفن همراه بر ایمنی ترافیکی: بررسی متدولوژی‌های مطالعه

http://hsr.mui.ac.ir/index.php/jhsr/article/view/380

پدیده استفاده از تلفن همراه در هنگام رانندگی توسط پژوهشگران زیادی مورد مطالعه قرار گرفت و تأثیر منفی آن بر ایمنی ترافیکی در مطالعه‌های گوناگون نشان داده شد. با این حال، چارچوب‌های پژوهشی که بر اساس آن چنین یافته‌هایی ارایه شدند، مورد بحث و نقد پژوهشگران است. در این مقاله چارچوب‌های متدولوژیک این مطالعه‌ها مورد بررسی قرار گرفت. مقاله‌های منتشر شده در شش مجله نمایه شده در ISI web of knowledge در حوزه‌های بهداشت و سلامت، ایمنی، حمل و نقل و علوم رفتاری بر اساس شش کلید واژه مرتبط با موضوع ایمنی ترافیکی و تلفن همراه جستجو شد. 13 مقاله با میانگین ارجاع 3/8 و بر اساس دسترسی به متن کامل از مجموع مقاله‌ها استخراج شد و مورد بررسی قرار گرفت.  سه رویکرد اصلی در تعیین تأثیر استفاده از تلفن همراه بر ایمنی ترافیکی در مقاله‌ها قابل شناسایی است. رویکرد اپیدمیولوژیک توسط متخصصین پزشکی اجتماعی و اپیدمیولوژیست‌ها، رویکرد مطالعه‌های شبیه‌سازی توسط متخصصین ارگونومی، ایمنی و ترافیک و رویکرد روان‌شناختی توسط متخصصین نورسایکولوژی، روان‌شناسی شناختی و روان‌شناسی کاربردی مورد استفاده قرار گرفت. هر کدام از رویکردها دارای محدودیت‌های متدولوژیک در بررسی این پدیده هستند. تمامی رویکردها تأثیر استفاده از تلفن همراه بر افزایش ریسک تصادفات رانندگی را تأیید کردند. با این حال، تعمیم کلی یافته‌های مطالعه‌ها به دلیل محدودیت‌های متدولوژیک مربوط به هر کدام از رویکردها دشوار است. نقاط قوت و ضعف هر کدام از متدولوژی‌ها مورد بحث قرار گرفت.

Title: تغذیه در حاملگی، شیردهی و یائسگی

http://hsr.mui.ac.ir/index.php/jhsr/article/view/642

سلامت و بهداشت زنان همواره یکی از مسایل مهم بهداشتی در جوامع بشری به شمار می‌رود. زنان در طول عمر خود از مراحل فیزیولوژیک حاملگی و شیردهی گذر می‌کنند و سپس در سنین میان‌سالی به مرحله یائسگی می‌رسند. هر کدام از این مراحل با یک سری تغییرات فیزیولوژیک همراه است. یکی از مواردی که برای حاملگی و شیردهی موفق و مرحله یائسگی بی‌خطر حایز اهمیت است، توجه به تغذیه و رژیم غذایی است. تغذیه در سراسر زندگی فرد مهم است، اما در هیچ یک از مراحل زندگی اهمیت آن به اندازه زمان حاملگی و شیردهی نیست. به ویژه امروزه با تغییراتی که در کیفیت غذاها و فراوری آن‌ها و نیز سبک زندگی حاصل شده است، لزوم توجه به آن را در ارتباط با سلامت کودک و ابعاد مختلف آن دو چندان می‌سازد. با افزایش سن و کاهش قدرت باروری در زنان و بروز یائسگی که با اثرات کوتاه و طولانی مدت بر سلامتی همراه است، چاقی و اضافه وزن عامل خطر عمده برای بسیاری بیماری‌های مزمن مانند بیماری‌های قلبی- عروقی، سرطان‌ها و پوکی استخوان محسوب می‌شود و با بسیاری از مسایل بهداشتی نیز مرتبط است. بنابراین با دریافت رژیم غذایی مناسب، سلامت زنان می‌تواند در سراسر عمر حفظ گردد.

Title: ارتقای بهداشت فردی در محیط کار بر اساس رهنمودهای بهداشتی اسلام

http://hsr.mui.ac.ir/index.php/jhsr/article/view/547

سلامتی نیاز اساسی انسان و لازمه انجام وظایف دینی می‌باشد. اسلام بر اساس ارزش وجودی انسان و ضرورت حیات، سلامت و کرامت او، رهنمودهایی برای شیوه زندگی و کار سالم و پیش‌گیری و دوری از گناه ارایه کرده و پاداش و جزا را نیز به عنوان پشتوانه آن در نظرگرفته است تا فرد بتواند مسیر تکاملی خود را بهتر طی کند.   مطالعه توصيفي- استنادی حاضر به بررسی موضوعی رهنمودهای دینی درباره بهداشت فردی در محیط کار بر اساس بیان مستقیم یا برداشت‌ها از منابع در دسترس شامل تفاسير قرآن و كتاب‌هاي حديثی معتبر پرداخت.    خداوند پاكيزگان‌ را دوست‌ دارد. با تمام توان تلاش كنيد تا پاكيزه باشيد؛ چرا که خداوند اسلام را بر پاكيزگي بنيان نهاده است. غسل و مسواک زدن و بوی خوش بر هر مسلمانی لازم است. ریش‌های خود را شانه و ناخن‌های خود را کوتاه کنید. لباس‌ پنبه‌اي‌ بپوشيد. پياده‌روي و شنا و نگاه كردن به سبزه سبب شادابي است. مصرف مشروبات الکلی و قمار حرام است. بر شما از پرخوری، پرخوابی، تنبلی و بی‌کاری و ضعف ایمان می‌ترسم. به رعایت میانه‌روی در امور، سفارش شده است. پیروی از آموزه‌های پیامبر (ص) و عترت پاک او به عنوان الگوهای عینی اسلام در رعایت بهداشت فردی، از ساده‌ترین کار مانند شستشوی دست تا استحمام کل بدن، آراستگی و پاکیزگی، نظافت مو و ناخن، لباس و پوشش‌ مناسب، فعالیت جسمانی، خودداری از مصرف مواد زیان‌آور و رفتارهای خطرناک می‌تواند در حفظ سلامتی و ارتقای سلامت کارگران مورد بهره‌برداری قرار گیرد.

Title: بررسی ویژگیهای بهداشت محیطی شهر سالم در آموزه های دینی

http://hsr.mui.ac.ir/index.php/jhsr/article/view/548

شهر مهم‌ترین مصرف کننده منابع طبیعی و همچنین تولید کننده بیشترین حجم مواد زاید، فضولات و آلودگی‌ها است و دارای اثرات منفی زیادی نیز بر زندگی انسان، طبیعت و ساختار شهرها می‌باشد. شهر سالم محیط اجتماعی و فیزیکی است که منابع و امکانات لازم برای زندگی و انجام وظایف و توسعه حداکثری توانایی‌های انسان را فراهم کند. شهر سالم باید بر اساس توجه به کرامت انسان، پاکیزگی و بهداشت محیط، امنیت و ایمنی، حوادث غیر مترقبه، آلودگی‌ها، ،حفظ ارتباط با گذشته، زیبایی و تذکر معنویت شکل بگیرد. در مقاله حاضر به برخی ویژگی‌های بهداشت محیطی شهر سالم در آموزه‌های دینی پرداخته شد. فراهم آوردن فضای پاک و سالم برای زندگی و فعالیت انسان، وظیفه دینی است و تمام جنبه‌هاي‌ زندگي‌ انساني‌ از جمله توجه به ‌بهداشت و پاکیزگی محیط، اعتدال و میانه‌روی در فعالیت‌های انسانی، برنامه‌ها و طرح‌های شهری مطابق با حفاظت محیط زیست، افزایش فضای سبز و تماس انسان با طبیعت، آبادانی زمین بر اساس تعادل طبیعی، توجه به طهارت روانی و معنوی در استفاده بهینه و مناسب از امکانات در دسترس و همچنین کاهش آلودگي‌ها و عوامل مؤثر در اتلاف هزینه و وقت ‌را در برمی‌گیرد

Title: مطالعه سبک زندگی سالم در سالمندان شهر اصفهان: یک مطالعه مقطعی

http://hsr.mui.ac.ir/index.php/jhsr/article/view/269

مقدمه: سبك زندگي در دوران سالمندی، با مرگ و میر و ناتوانی بسیاری از بیماری‌ها از جمله بیماری‌های عروق کرونر، بیماری‌های قلبی و سرطان‌ها مرتبط است. بر همین اساس، این مطالعه با هدف بررسی سبک زندگی سالم در سالمندان کانون بازنشستگان شهر اصفهان انجام شد.   روش‌ها: در این مطالعه مقطعی، 250 نفر از سالمندان عضو کانون بازنشستگان استان اصفهان مورد مطالعه قرار گرفتند. مشخصات دموگرافیک، آگاهی و سبک زندگی سالم سالمندان به وسیله پرسش‌نامه‌ای دارای روایی و پایایی مورد تأیید، جمع‌آوری گردید. در تجزیه و تحلیل داده‌ها از نرم‌افزار 15SPSS و آزمون‌های Indipendent T و Dipendent Pearson استفاده شد. یافته‌ها: از مجموع 250 نفر شرکت کننده در مطالعه، 140 نفر (56 درصد) آنان را مردان تشکیل می‌دادند. میانگین سنی سالمندان مورد مطالعه 55/3 ± 04/67 سال بود. میانگین نمره پرسش‌نامه سبک زندگی سالم در سالمندان 22/153 با انحراف معیار 89/14 بود. میانگین نمره سبک زندگی سالم سالمندان در دو جنس، اختلاف معنی‌داری نشان نداد (001/0 > P). نتایج آزمون Dipendent Pearson حاکی از وجود ارتباط معنی‌دار بین میانگین نمره حیطه‌های مختلف سبک زندگی سالم با نمره آگاهی و نمایه توده بدن بود. نتیجه‌گیری: سطح تحصیلات و آگاهی از مهم‌ترین عوامل تأثیرگذار بر سبک زندگی سالم در سالمندان می‌باشد. به نظر می‌رسد که افزایش آگاهی مرتبط با سبک زندگی سالم و بیماری‌های مزمن در افراد سالمند، می‌تواند به سالمندان در درک پیامدهای بالقوه سلامت ناشی از عملکرد خود و نیز تشویق آن‌ها به تغییر در شیوه زندگی مؤثر باشد.

Title: مطالعه تطبیقی الزامات سیستم مدیریت ایمنی فرایند (PSM) با سیستم‌های 18001OHSAS (OH&S) و HSE و امکان‌سنجی استقرار آن در یکی از شرکت‌های پتروشیمی کشور

http://hsr.mui.ac.ir/index.php/jhsr/article/view/289

مقدمه: در صنایع فرایندی، تولید، ذخیره، حمل، استفاده و دفع مواد شیمیایی بالقوه خطرناک بوده و پتانسيل رخداد حوادث فاجعه‌بار در آن‌ها بالا است. جهت ارتقای ایمنی در این صنایع، استقرار سیستم مدیریت ایمنی فرایند (Process safety management یا PSM) یکی از مؤثرترین اقدامات به شمار می‌رود. مطالعه حاضر با هدف، تعيين درجه انطباق الزامات سيستم‌های [ Occupational health and safety assessment seriesیا (OH&S) 18001 OHSAS] و (HSE-MS  یا Health safety and environment managementsystems) با سيستم PSM و همچنين بررسی ميزان اجرای الزامات PSM و امکان‌سنجی استقرار آن در يکی از واحدهای پتروشيمی کشور انجام شد. روش‌ها: طی يک بررسی کتابخانه‌ای در مرحله اول مطالعه، کليه الزامات سيستم‌های PSM، OH&S و HSE-MS استخراج گردید. سپس کليه الزامات این سه سیستم تطبيق داده شد و درجه انطباق آن‌ها به صورت درصد مشخص گرديد. در مرحله بعد، درجه اجرای الزامات PSM و امکان استقرار کامل آن بر اساس راهنمای مميزی PSM سازمان OSHA مورد بررسی قرار گرفت. اطلاعات مورد نياز از طريق بررسی مستندات، مصاحبه با کارکنان و بررسی شواهد میدانی جمع‌آوری شد. یافته‌ها: مقايسه الزامات سه سيستم مديريتی مورد بررسی نشان داد، سيستم PSM با OH&S در 8 الزام و با HSE-MS در 9 الزام مشترک است. 5 الزام شامل اطلاعات ایمنی فرایند، ملاحظات ایمنی پیش از راه‌اندازی، یکپارچگی مکانیکی، مجوز کار گرم و اسرار تجارت، ويژه سيستم مديريت ايمنی فرايند می‌باشد. میزان الزامات مشترک PSM با سیستم‌های OH&S و HSE-MS به ترتیب برابر با 14/57 و 28/64 درصد تعیین شد. همچنين، نتايج اين بررسی نشان داد که 4/67 درصد الزامات سيستم PSM در صنعت مورد بررسی استقرار يافته است. نتیجه‌گیری: با بازنگری در برخی از مستندات سيستم‌های مديريتی OH&S و HSE-MS و درج الزامات PSM در آن‌ها و تهيه ساير مستندات اختصاصی مربوط به PSM، می‌توان نسبت به استقرار سيستم مديريت ايمنی فرايند در صنعت مورد مطالعه اقدام نمود. با توجه به تشابه شرایط صنعت مورد مطالعه با دیگر صنایع پتروشیمی در سطح کشور، می‌توان نتایج مطالعه حاضر را به این صنایع تعمیم داد.

Title: بررسی میزان کارایی تالاب مصنوعی زیر سطحی در کاهش ازت آمونیاکی و فسفر از فاضلاب مصنوعي مشابه خانگي در مقیاس آزمایشگاهی

http://hsr.mui.ac.ir/index.php/jhsr/article/view/328

مقدمه: تالاب‌های مصنوعی شبيه‌سازي فرایندهای پالایش در تالاب‌های طبیعی مي‌باشند که به طور مؤثری آلودگی‌های مختلف را از فاضلاب‌ها حذف می‌کنند. هدف از تحقيق حاضر، امكان‌سنجي استفاده از تالاب مصنوعي زير سطحي با جریان افقی در حذف ازت آمونياكي و فسفر از فاضلاب مصنوعي مشابه خانگي در مقياس آزمايشگاهي بود. روش‌ها: در این مطالعه، یک تالاب مصنوعی زیر سطحی با جریان افقی در مقیاس آزمایشگاهی ساخته شد. زمان ماند برابر 6 روز و جریان فاضلاب به صورت ثقلی و پیوسته بود. فاضلاب مصنوعی مشابه فاضلاب خانگی به عنوان پساب ورودي استفاده گرديد. نسبت COD/N/P به صورت 2/32/100 در نظرگرفته شد. مواد مورد استفاده در لایه‌بندی بستر شامل ذغال چوب، پوکه معدنی، شن درشت و خاک مناسب رشد گیاه بود که با توجه به مشخصات بستر شیب 2 درصد و تخلخل 40-30 درصد بود. عمق سیستم با توجه به عمق طراحی برای نی معمولی 7/0 متر انتخاب شد و میزان جریان مورد تصفیه با استفاده از فرمول Darcy برای لایه‌های متخلخل 50 لیتر در روز به دست آمد. دو گونه گیاهي علف دم گربه‌ای و نی معمولی (فراگماتیس استرالیس) در تالاب کاشته شدند. از روش‌های مناسب آماری و نرم‌افزار 12SPSS برای تجزیه و تحلیل نتایج استفاده شد. یافته‌ها: کارایی رآکتور تالاب مصنوعی زیر سطحی با استفاده از آزمون Paired t با اطمینان 95 درصد در حذف ازت آمونیاکی و فسفر به ترتیب 06/35 و 31 درصد بود. حداکثر و حداقل تغییرات کل جامدات معلق نیز به ترتیب 37 و 20 میلی‌گرم در لیتر به دست آمد. نتیجه‌گیری: کارایی رآکتور تالاب مصنوعی زیر سطحی با بستر ذغال و پوکه معدنی با استفاده از گیاهان علف دم‌ گربه‌ای و نی معمولی در حذف ازت آمونیاکی و فسفر مناسب بوده و می‌تواند با فراهم کردن شرایط مناسب راهبری و نگهداری به عنوان یک جایگزین دوست‌دار محیط زیست به جای سایر سیستم‌های مکانیکی تصفیه فاضلاب باشد.

Title: اندازه‌گیری ابعاد آنتروپومتریک استاتیک (تن‌سنجی ایستا) و بررسی معادلات رگرسیونی بین آن‌ها در یک جمعیت دانشجویی

http://hsr.mui.ac.ir/index.php/jhsr/article/view/495

مقدمه: برای ایجاد تناسب ابعادی بین انسان و ابزار یا محیط پیرامون او وجود بانک اطلاعات آنتروپومتری ضروری است. هدف از انجام این مطالعه، سنجش ابعاد بدن و بررسی معادلات رگرسیون بین آن‌ها در یک جامعه دانشجویی برای تأسیس بانک اطلاعات آنتروپومتریک بود. روش‌ها: مطالعه مقطعی حاضر بر روی 194 دانشجوی مشغول به تحصیل در دانشکده بهداشت و تغذیه دانشگاه علوم پزشکی شیراز (5/50 درصد دختر و 5/49 درصد پسر) انجام شد. ابزار جمع‌آوری داده‌ها در این مطالعه پرسش‌نامه ويژگي‌هاي دموگرافيک و ابزار سنجش ابعاد بدن شامل استودیومتر، کولیس، متر نواری و ترازو بود. تجزیه و تحلیل داده‌های مطالعه با استفاده از نرم‌افزار 16SPSS انجام گرفت. مقدار P کمتر از 05/0 به عنوان سطح معنی‌داری در نظر گرفته شد. یافته‌ها: میانگین سن دانشجویان پسر و دختر به ترتیب 20/2 ± 09/22 و 8/1 ± 07/22 سال به دست آمد. میانگین قد این دو گروه نیز به ترتیب 73/4 ± 52/177 و 73/4 ± 10/161 سانتی‌‌متر به دست آمد. ارتفاع چشم، شانه و آرنج در دو حالت ایستاده و نشسته ارتباط معنی‌‌دار با ارتفاع قد داشت (05/0 > P). نتیجه‌گیری: بانک اطلاعاتی آنتروپومتریک تأسیس شده می‌‌تواند در طراحی وسایل این جامعه هدف مورد استفاده قرار گیرد. یافته‌های رگرسیونی نشان داد که ابعاد ارتفاع چشم، شانه و آرنج در دو حالت ایستاده و نشسته دارای همبستگی بالایی با ارتفاع قد می‌باشند و بنابراین می‌توان از روی ارتفاع قد، ابعاد یاد شده را با کمترین اندازه‌گیری برآورد نمود.

Title: بررسی پیشگویی کننده‌های مراقبت‌های بهداشت دهان و دندان در مادران باردار شهر اراک بر اساس مدل اعتقاد بهداشتی

http://hsr.mui.ac.ir/index.php/jhsr/article/view/494

مقدمه: رعایت رفتارهای بهداشت دهان و دندان در گروه پر مخاطره مادران باردار به دلیل حفظ سلامت خود و جنین آن‌ها اهمیت زیادی دارد. الگوی اعتقاد بهداشتی از الگوهای روان‌شناسی است که تلاش می‌کند تا رفتارهای بهداشتی را با تمرکز بر اعتقادات و نگرش‌های افراد توصیف نماید. از این رو پژوهش حاضر با هدف تعیین پیشگویی ‌کننده‌های مراقبت‌های بهداشت دهان و دندان در مادران باردار شهر اراک بر اساس مدل اعتقاد بهداشتی انجام شد. روش‌ها: در این مطالعه مقطعی تحلیلی، 340 نفر از مادران باردار شهر اراک با روش نمونه‌گيري تصادفي ساده از مراکز بهداشتی درمانی انتخاب شدند. مقياس مربوط به عوامل مدل اعتقاد بهداشتی (حساسيت، شدت، تهدید، منافع، موانع و خودکارامدی درک‌شده و راهنماهای عمل) و عملکرد در خصوص مراقبت‌های بهداشت دهان و دندان از طریق مصاحبه و مشاهده سنجیده شد. در نهایت اطلاعات مورد تجزیه و تحلیل قرار گرفت. يافته‌ها: از بين متغيرهاي مدل اعتقاد بهداشتي، خودکارامدي و موانع درک‌شده بیشترین عامل پيش‌بيني کننده رفتار بهداشتي دهان و دندان در مادران باردار بودند. میانگین نمره عملکرد مادران در خصوص انجام مراقبت‌های بهداشت دهان و دندان 7/9 ± 1/43 بود. در این پژوهش بین حساسیت درک‌شده و عملکرد ( 44/0 = r)، شدت درک‌شده و عملکرد (51/0 = r) و خودکارامدی و عملکرد (61/0 = r) ارتباط مثبت مشاهده شد (05/0 > P). این در حالی است که بین موانع درک‌شده در خصوص انجام رفتارهای مراقبت بهداشت دهان و دندان و عملکرد مادران در این زمینه ارتباط معکوس مشاهده شد (65/0- =r). متغیرهای فوق 8/46 درصد از واریانس رفتارهای مراقبت بهداشت دهان و دندان را بیان نمودند. نتيجه‌گيري: مطالعه حاضر کارایی الگوی اعتقاد بهداشتی را در پیشگویی اتخاذ مراقبت‌های بهداشت دهان و دندان در مادران باردار نشان داد. از این رو این الگو می‌تواند به عنوان چارچوبی جهت طراحی و اجرای مداخلات آموزشی جهت کاهش پوسیدگی دندان استفاده شود و در دوران بارداری به حفظ سلامت مادر و کودک کمک نماید.

Title: بررسی ارتباط اعتیاد به اینترنت با سیگار و وضعیت تحصیلی در دانشجویان دانشگاه علوم پزشکی لرستان

http://hsr.mui.ac.ir/index.php/jhsr/article/view/474

مقدمه: گسترش روزافزون استفاده از اینترنت، عدم استفاده صحیح از آن و جستجوهای غیر هدفمند نه تنها دانشجویان را از رسالت اصلی اینترنت در دانشگاه دور می‌کند، بلکه با اتلاف زمان می‌‌‌تواند تأثیر سویی بر وضعیت تحصیلی آنان بگذارد. این مطالعه با هدف، تعیین ارتباط اعتیاد به اینترنت با وضعیت تحصیلی و مصرف سیگار در دانشجویان دانشگاه علوم پزشکی لرستان انجام شد. روش‌ها: مطالعه حاضر به صورت مقطعي و بر روی 368 نفر از دانشجویان كاربر اینترنتی دانشگاه با نمونه‌گیری چند مرحله‌ای صورت گرفت. جمع‌آوری داده‌ها با استفاده از پرسش‌نامه اعتیاد به اینترنت Young و آزمون‌های Paired t، ANOVA، Tukey، Mann-Whitney، Kruskal-Wallis، ضریب همبستگی Spearman و Eta با سطح اطمینان 05/0 انجام گردید و توسط نرم‌افزار 16SPSS مورد تجزیه تحلیل قرار گرفت. یافته‌ها: از تعداد 368 نفر دانشجو، 163 نفر (3/44 درصد) مرد و 205 نفر (7/55 درصد) زن بودند. 3/63 درصد از افراد، وابستگی خفیف و 3/7 درصد وابستگی شدید به اینترنت را نشان دادند و تمام افراد دارای وابستگی شدید به اینترنت، افت تحصیلی داشتند. یافته‌ها ارتباط مستقیم معنی‌داری بین افت تحصیلی و استعمال سیگار با میانگین نمره اعتیاد به اینترنت و همچنین ارتباط مستقیم بین مقطع تحصیلی و سطح کاربری اینترنتی و ارتباط معکوسی بین معدل تحصیلی و سطح کاربری اینترنتی (01/0 > P) را نشان داد. نتیجه‌گیری: شیوع وابستگی به اینترنت در این مطالعه نگران کننده بود و به نظر می‌رسد زمان مداخله برای پیش‌گیری از اثرات جسمانی و روانی اعتیاد به اینترنت با فرهنگ‌سازی استفاده مناسب از آن فرا رسیده است.

Title: مهارت‌های مطالعه در دانشجویان دانشگاه علوم پزشکی گلستان یک نیاز فراموش شده

http://hsr.mui.ac.ir/index.php/jhsr/article/view/478

مقدمه: پیشرفت تحصیلی به دانش و کاربرد مؤثر مهارت‌های مطالعه بستگی دارد. این مطالعه با هدف بررسی مهارت‌های مطالعه در بین دانشجویان دانشگاه علوم پزشکی گلستان صورت گرفت.  روش‌ها: در یک مطالعه توصیفی مقطعی، تعداد 558 دانشجو در سال تحصیلی 88-1387 از طریق سرشماری وارد مطالعه شدند. ابزار جمع‌آوری داده‌ها سیاهه مهارت‌های مطالعه کانگاس بود. تجزیه و تحلیل داده‌ها از طریق آمار توصیفی و آزمون‌های آماری Independent t، آنالیز واریانس و ضریب همبستگی Pearson انجام گرفت. یافته‌ها: میانگین و انحراف معیار نمرات 39/0 ± 45/2 از 4 بود. مهارت‌های مطالعه در 4/44 درصد افراد ضعیف، 4/53 درصد متوسط و 2/2 درصد خوب بود. بین مهارت‌های مطالعه و جنسیت ارتباط معنی‌داری مشاهده شد (05/0 > P). همبستگی مثبتی نیز بین معدل تحصیلی و مهارت‌های مطالعه مشاهده شد (13/0 = r). میانگین نمره مهارت‌های مطالعه دانشجویان جدیدالورود نسبت به دانشجویان ترم‌های بالاتر به طور معنی‌داری بیشتر بود (05/0 > P). نتیجه‌گیری: اکثریت دانشجویان مورد بررسی دارای مهارت‌های مطالعه ضعیف و متوسطی بودند. ضرورت آموزش مهارت‌های مطالعه به دانشجویان در حد آموزش کارگاهی و یا به شکل ارایه واحد درسی در ابتدای ورود به دانشگاه محسوس است.

Title: بررسی كيفـيت ‌زندگي و ارتباط آن با ویژگی‌های بیماری و اطلاعات دموگرافیک در مبتلایان به سـرطان مراجعه کننده به بیمارستان انکولوژی دانشگاه علوم پزشکی اصفهان

http://hsr.mui.ac.ir/index.php/jhsr/article/view/568

مقدمه: در دهه‌های ‌اخیر، کیفـیت ‌زندگی به ویژه در بیماران ‌مبتلا ‌به ‌سرطان، به ‌دلیل ‌شرایط نامساعد جسمی ‌و روانی‌ این بیماران، همواره مورد توجه متخصصان ‌بالینی و پژوهشگران ‌علوم ‌بهداشتی بوده ‌است. از طرفی، مفهوم ‌کیفیت ‌زندگی و ارتباط آن با ویژگی‌های ‌بیماری ‌سرطان ‌‌و ‌اطلاعات ‌دموگرافیک ‌از جمله ‌پارامترهای ‌قدرتمند ‌در ‌راستای ‌راهنمایی، حفظ‌ و پیشبرد‌ سلامت‌ و تندرستی ‌بیماران ‌در جوامع‌ و فرهنگ‌ها می‌باشد. از این‌رو انجام‌ مطالعات ‌عمیق در این ‌حوزه‌، اطلاعات‌ با ارزشی ‌را‌ در اختیار پرسـتاران قرار می‌دهد و غنای ‌مداخلات ‌بهداشتی- درمانی و بهبود کیفیت ‌خدمات‌ پرستاری ‌را ‌به ‌دنبال‌ خواهد ‌داشت. به همین دلیل، مطالعه حاضر با هدف بررسی ‌كيفـيت‌ زندگي و ارتباط آن با ویژگی‌های‌ بیماری و اطلاعات‌ دموگرافیک در مبتلایان ‌به ‌سرطان مراجعه ‌کننده به بیمارستان ‌انکولوژی‌ دانشگاه ‌علوم ‌پزشکی ‌اصفهان انجام شد. روش‌ها: مطالعه‌ حاضر از نوع توصیفی- تحلیلی بود که به شکل‌ مقطعی اجرا گردید. نمونه ‌مورد پژوهش در این‌ مطالعه شامل 315 نفر از مبتلایان ‌به ‌سرطان‌ بود که با روش‌ نمونه‌گیری‌ آسان وارد مطالعه ‌شدند. ابزار گردآوری ‌‌اطلاعات‌ شامل ‌پرسش‌نامه سـه ‌قسمتی ‌متشکل‌ از سؤالات ‌مربوط ‌به ویژگی‌های ‌‌بیماری، اطلاعات ‌‌دموگرافیک ‌و پرسش‌نامه ‌اسـتاندارد كيفيت ‌زندگي 36-SF بود. داده‌ها پس‌ از ‌جمع‌آوری ‌با استفاده از نرم‌افزار آماری 18SPSS و روش‌های‌آمار توصیفی (توزيع ‌درصد فراواني، ميانگين، انحراف ‌معيار) و استنباطی (Chi-square test، Cho Proof، Kruskal-Wallis، Mann-Whitney U، Pearson و Spearman correlation coefficient) و با ضریب ‌اطمینان 99 درصد مورد تجزیه ‌و تحلیل ‌قرار گرفت.   یافته‌ها: کیفـیت ‌زندگی اغلب (17/63 درصد) واحدهای‌ مورد پژوهش متوسـط بود. بین نوع ‌سرطان و شدت ‌درد ناشی از بیماری با کیفـیت‌ زندگی ‌بیماران ‌مبتلا ‌به ‌سرطان، ارتباط آماری‌ معنی‌داری وجود داشت (050/0 P <). ‌بین‌کیفـیت ‌زندگی ‌بیماران‌ مبتلا‌ به ‌سرطان ‌با تعداد‌ جلسات شیمـی‌‌درمانی ارتباط ‌آماری ‌معنی‌داری مشاهده شد؛ به‌ طور‌ی ‌که ‌بیماران با تعداد ‌جلسات بیشتر شیمی‌درمانی، از کیفـیت ‌زندگی بهتری برخوردار بودند. ارتباط آماری ‌معنی‌داری بین متغیرهای ‌دموگرافیک و ویژگـی‌های ‌بیماری نظیر سن، جنس، وضعیت‌ اشتغال، پذیرش ‌یا‌ انکار ‌بیماری، تعداد ‌اعضای ‌خانواده، مرحله ‌سرطان و طول ‌مدت ‌زمان ‌سپری‌ شده ‌از تشخیص بیماری با کیفـیت ‌زندگی وجود نداشت. همچنین نتایج مطالعه ‌حاضـر ‌حاکی از معنی‌دار شدن ارتباط بین وضعیت تأهل با کیفـیت ‌زندگی (018/0 = P)، میزان درامد ماهیانه با کیفـیت ‌زندگی (001/0 = P) و وضعیت تحصیلات با کیفـیت ‌زندگی (004/0 = P) بود. نتیجه‌گیری: برخی ویژگی‌های ‌سرطان و شاخص‌های‌ دموگرافیک، کیفـیت ‌زندگی ‌بیماران را متأثر می‌سازد و ارتباط محسوسی بین آن‌ها وجود دارد. از این‌رو، توجه به این مقوله از سوی‌ کادر درمانی در اعتلای ‌کیفـیت‌ زندگی بیماران بسیار حایز اهمیت ‌می‌باشد. در این میان بر نقش‌ پرسـتاران ‌انکولوژی‌ تأکید می‌شود، چرا که پرسـتاران به ‌عنوان یکی از اعضای تیم‌ درمان، نقش ‌مهمی در تشخیص، درمان و مراقبت از بیماران ‌مبتلا به سرطان بر عهده‌ دارند. پیشنهاد می‌شود پرسـتاران‌ در زمینه ‌عوامل ‌مؤثر بر کیفـیت‌ زندگی، آموزش‌های ‌لازم را دریافت ‌کنند تا با بهره‌مندی از این آموزش‌ها، با نگرش‌ منطقی‌ و‌ دانشی ‌غنی در بهبود ‌کیفـیت ‌زندگی ‌بیماران ‌مبتلا ‌به ‌سرطان، ‌بیش ‌از پیش مشارکت و ایفای نقش نمایند. واژه‌های ‌کلیدی: كيفيـت ‌زندگي، ویژگی‌های ‌سرطان، اطلاعات ‌دموگرافیک،‌ سرطان‌، پرستاری

Title: بررسی وضعیت افسردگی دانشجویان دانشگاه‌های شهر بردسیر استان کرمان

http://hsr.mui.ac.ir/index.php/jhsr/article/view/599

مقدمه: متأسفانه شیوع برخی از مشکلات روحی و روانی در بین دانشجویان به ویژه افسردگی موجب مشکلات زیادي براي این گروه تأثیرگذار شده است. بررسی چنین مسأله مهمی می‌تواند تصمیم‌گیرندگان جامعه را در برنامه‌ریزي و پیشگیري از آن یاري نماید و دانشجویان جامعه را که اساسی‌ترین سرمایه یک کشور به حساب می‌آیند را سالم، شاداب و توانمند به عرصه‌هاي کار و فعالیت هدایت نماید. اين پژوهش با هدف تعيين ميزان افسردگی و عوامل مرتبط با آن در دانشجویان دانشگاه‌های بردسیر صورت گرفت.   روش‌ها: اين پژوهش يك مطالعه توصیفی- تحلیلی است كه در سال 90-1389 انجام شد. جامعه پژوهشی در این مطالعه دانشجویان رشته‌‌هاي مختلف دانشگاه‌هاي دولتی، آزاد و پیام‌نور شهرستان بردسیر بود. حجم نمونه 300 نفر بود و نمونه‌گيري به روش تصادفی ساده با نمونه‌های در دسترس انجام شد. ميزان افسردگی دانشجویان با استفاده از پرسش‌نامه دو قسمتی شامل اطلاعات دموگرافیک و مرتبط با بیماری (20 سؤال) و پرسش‌نامه استاندارد افسردگی زونگ (20 سؤال) بر اساس مقیاس چهار درجه‌ای لیکرت اندازه‌گیری گردید. داده‌های جمع‌آوری شده با استفاده از نرم‌افزار 5/11SPSS و با استفاده از آزمون 2χ و آزمون Exact fisher مورد تجزیه و تحلیل قرار گرفت. یافته‌ها: 300 دانشجوی دختر (3/79 درصد) و پسر (7/20 درصد) با رعایت موازین اخلاقی پژوهش، آزادانه در این مطالعه شركت نمودند. امتياز افسردگی با عوامل دموگرافيك مانند جنس، سن، رتبه تولد، ابعاد خانوار، رشته تحصیلی، سن و تحصیلات و شغل مادر، سن، تحصیلات و شغل پدر، وضعيت طبیعی زندگی، وضعیت مسکن، درامد ماهیانه خانواده، رضایت از تحصیل و پیش‌بینی قبولی در کنکور مورد تجزيه و تحليل قرار گرفت. يافته‌هاي مطالعه نشان داد كه جنس ( 001/0 P <)، سطح تحصیلات مادر (03/0 = P)، رتبه تولد (02/0 = P)، رضایت از تحصیل (01/0 = P) و پیش‌بینی قبولی در کنکور (002/0 = P) با میزان افسردگی ارتباط معني‌داري داشت.  نتیجه‌گیری: با توجه به این که افسردگی به عنوان یکی از موضوعات مهم در بهداشت روان به شمار می‌رود و این که درصد چشمگیری از دانشجویان مورد مطالعه درجات متنوعی از افسردگی را داشتند، ضرورت انجام مداخلات آموزشی با بهره‌گیری از مدل‌های آموزش بهداشت و ارتقای سلامت با تمرکز بر بهداشت روان اجتناب‌ناپذیر است.

Title: تعیین عوامل مرتبط با تعداد تصادفات بر حسب شدت با استفاده از رگرسیون پواسون- لگ نرمال چند متغیره بیزی

http://hsr.mui.ac.ir/index.php/jhsr/article/view/604

مقدمه: برازش مدل‌های تک متغیره در بسیاری از تحقیق‌های گذشته برای تحلیل داده‌های تصادف به کار رفته است. با توجه به آن که متغیر شدت در این مطالعه‌ها می‏تواند بیش از یک سطح باشد، از این رو در این مقاله مدل پواسون- لگ نرمال چند‏ متغیره برای مدل‏سازی تعداد تصادفات بر حسب شدت استفاده شد. اگرچه برای مقایسه از مدل‌های تک متغیره نیز استفاده شد. روش‌ها: استنباط آماری پارامترهای مدل توسط رهیافت بیز و استفاده از روش نمونه‌گیری گیبز و الگوریتم متروپلیس- هستینگز انجام شد. داده‏های تصادف مربوط به تقاطع‏های شهر اصفهان بود. یافته‌ها: با وجود بیش پراکنش در دو سطح شدت و وجود همبستگی بین این دو سطح، مدل پواسون- لگ نرمال چند متغیره برازش بهتری را نسبت به بقیه مدل‌ها ارایه داد. همچنین اثر حجم کل ترافیک بر تصادفات خسارتی در تمامی مدل‌ها معنی‌دار بود، اما اثر حجم کل ترافیک چپ‌گردها بر تصادفات جراحتی و فوتی تنها در مدل پواسون تک متغیره معنی‌دار شد. بنابراین با فرض ثابت نگه داشتن بقیه متغیرهای توضیحی، انتظار می‏رود که افزایش حجم کل ترافیک باعث افزایش تصادفات خسارتی می‌شود. همچنین افزایش حجم کل ترافیک چپ‌گردها نیز باعث افزایش تصادفات جراحتی و فوتی در تقاطع‏ها می‏شود. به طور دقیق‌تر تحت مدل پواسون- لگ نرمال چند متغیره افزایش 1000 وسیله نقلیه در متوسط حجم کل ترافیک باعث افزایش 31 درصدی تصادفات خسارتی می‏شود. نتیجه‌گیری: بنابراین کاهش حجم کل ترافیک در مقوله کاهش هزینه تصادفات در دراز مدت پیش‌بینی می‏شود که بسیار مقرون به صرفه است

Title: تنظیم نقشه‌ جغرافیایی میزان بروز سرطان معده در ایران طی دوره پنج ساله با استفاده از روش پواسن کریگیدن (Poisson kriging) منطقه به منطقه

http://hsr.mui.ac.ir/index.php/jhsr/article/view/756

مقدمه: سرطان معده بالاترین شیوع را در میان سرطان‌های دستگاه گوارش در ایران دارد. بنابراین مطالعه حاضر برای تبیین توزیع جغرافیایی سرطان معده با روش دقیق پواسن کریگیدن (Poisson kriging) منطقه به منطقه در سطح کلیه شهرستان‌های ایران و شناسایی مناطق پرخطر انجام گرفت. روش‌ها: این مطالعه از نوع کاربردی- بوم‌شناسی بود و از داده‌های ثبت شده توسط اداره سرطان مرکز مدیریت بیماری‌های غیر واگیر وزارت بهداشت درمان و آموزش پزشکی استفاده شد. داده‌هایی که در سطح 336 شهرستان و در طول سال‌های 86-1382 جمع‌آوری شد، مورد تجزیه و تحلیل قرار گرفت. نرم‌افزار Spacestat برای برآورد پارامترها و نرم‌افزار 9.3ArcGIS برای نمایش برآوردها بر روی نقشه به کار گرفته شد. یافته‌ها: میانگین میزان بروز بر اساس روش پواسن کریگیدن منقطه به منطقه (19/5) و میانگین واریانس میزان بروز (72/0) برآورد گردید. بیشترین میزان بروز (36/16) با واریانس (01/1) مربوط به شهرستان دیوان‌دره و کمترین میزان بروز (12/0) با واریانس (67/0) مربوط به شهرستان سرباز برآورد شد. کمترین واریانس (01/0) مربوط به تهران و بیشترین واریانس (51/2) مربوط به کوهبنان به دست آمد. نتیجه‌گیری: با توجه به برازش مناسب روش پواسن کریگیدن منطقه به منطقه در مناطق ناهمگن فضایی، استفاده از این روش در نقشه‌بندی بیماری پیشنهاد می‌شود. این مطالعه نشان داد که در شهرستان‌های استان‌های اردبیل، مازندران و کردستان میزان بروز بیماری بیشتر است.

Title: بررسی عملکرد رانندگان موتور سیکلت در استفاده از کلاه ایمنی بر اساس مدل اعتقاد بهداشتی

http://hsr.mui.ac.ir/index.php/jhsr/article/view/574

مقدمه: صدمات ناشي از حوادث جاده‌اي يكي از چالش‌هاي بزرگ، اما ناديده گرفته شده در بهداشت عمومي است. با وجود این که کلاه ایمنی خطر جراحات وارده به سر را به میزان قابل توجهی کاهش می‌دهد، تعداد کمی از موتور سواران از کلاه ایمنی استفاده می‌کنند. این مطالعه به بررسی استفاده از کلاه ایمنی در رانندگان موتور سیکلت بر اساس مدل اعتقاد بهداشتی پرداخته است. روش‌ها: نوع مطالعه مقطعی و جمعیت مورد پژوهش 267 نفر از رانندگان موتور سیکلت در شهر تبریز بودند. ابزار گردآوری داده‌ها پرسش‌نامه‌ای شامل مشخصات دموگرافیک و سازه‌های مدل اعتقاد بهداشتی بود. داده‌ها با استفاده از نرم‌افزار 5/11SPSS و آزمون‌های آماری ضریب همبستگی Spearman و آنالیز واریانس (Repeated measures ANOVA) مورد تجزیه و تحلیل قرار گرفتند. یافته‌ها: میانگین سنی افراد مورد پژوهش 5/10 ± 6/30 و دامنه آن 65-15سال بود. 9/25 درصد افراد مورد پژوهش اعلام کردند که همیشه از کلاه ایمنی استفاده می‌کنند. 22 درصد هرگز از کلاه ایمنی استفاده نمی‌کردند. 19 درصد سابقه تصادف در حین رانند گی با موتور سیکلت را داشتند. طبق نتایج، افراد مورد پژوهش بیشترین میانگین نمره را در منافع درک شده به دست آورده بودند (1/87 از 100 نمره). بین تمام متغیرهای مدل اعتقاد بهداشتی با عملکرد رانندگان در استفاده از کلاه ایمنی رابطه معنی‌داری وجود داشت (001/0 > P). نتیجه‌گیری: یافته‌های این بررسی نشان داد که میزان استفاده از کلاه ایمنی پایین است. در برنامه‌های آموزشی جهت ارتقای استفاده از کلاه ایمنی، می‌توان مدل اعتقاد بهداشتی را به کار گرفت.

Title: ارتباط‌سنجی حمایت اجتماعی درک‌شده و عوامل مرتبط با آن با کنترل گلیسمیک در بیماران مبتلا به دیابت نوع 2 مراجعه‌کننده به بیمارستان‌های تابعه دانشگاه علوم پزشکی تهران

http://hsr.mui.ac.ir/index.php/jhsr/article/view/598

مقدمه: عوامل متعددی در کنترل بیماری دیابت ایفای نقش می‌نمایند. از جمله عوامل اجتماعی مؤثر در کنترل این بیماری، حمایت اجتماعی درک‌شده از منابعی مانند دوستان و خانواده می‌باشد. برای بررسی ارتباط انواع منابع حمایت اجتماعی درک‌شده و عوامل مرتبط آن با شاخص استاندارد کنترل دیابت، مطالعه‌ای در این زمینه طراحی و اجرا گردید. روش‌ها: پژوهش حاضر يك مطالعه توصیفی- تحلیلی است که با هدف ارتباط‌سنجی بین حمایت اجتماعی درک‌شده از دوستان و خانواده و عوامل مرتبط با آن با شاخص کنترل دیابت (C1HbA) انجام شد. این مطالعه روی 317 بیمار مبتلا به دیابت نوع 2 مراجعه‌کننده به بیمارستان‌های دانشگاه علوم پزشکی تهران در طول 6 ماه اول سال 1390 انجام شد. ابزار جمع‌آوري اطلاعات این مطالعه، پرسش‌نامه‌ای مشتمل بر سه بخش بود. بخش اول شامل اطلاعات دموگرافیک و مرتبط با بیماری افراد، بخش دوم ابزار استاندارد اندازه‌گیری حمایت اجتماعی درک‌شده بیماران مبتلا به دیابت از خانواده (20 سؤال) و بخش سوم شامل ابزار استاندارد اندازه‌گیری حمایت اجتماعی درک‌شده بیماران مبتلا به دیابت از دوستان (20 سؤال) بود. در نهايت اطلاعات جمع‌آوري شد و توسط نرم‌افزار 5/11SPSS با استفاده از آزمون‌هاي توصیفی (میانگین، میانه و دامنه میان چارکی) و استنباطی (Mann-Whitney و Kruskal-Wallis) مورد تجزيه و تحليل قرار گرفت. یافته‌ها: یافته‌های مطالعه نشان داد که متغیرهای گروه سنی (01/0 P =)، شاخص توده بدنی (05/0 P =)، جنس (05/0 P =)، تأهل (001/0 P <)، طول مدت ابتلا به دیابت (001/0 P <)، سطح تحصیلات (006/0 P =) و شغل (001/0 P <) در حمایت درک‌شده از دوستان با C1HbA ارتباط معنی‌دار آماری داشت. همچنین متغیرهای گروه سنی (001/0 P <)، شاخص توده بدنی (001/0 P <)، جنس (001/0 P <)، تأهل (001/0 P <)، طول مدت ابتلا به دیابت (001/0 P <)، سطح تحصیلات (004/0P =) و شغل (03/0= P) در حمایت درک‌شده از خانواده با C1HbA ارتباط معنی‌دار آماری نشان داد. نتیجه‌گیری: حمایت درک‌شده از خانواده و دوستان با کنترل گلیسمیک در دیابت ارتباط داشت. بنابراین ضرورت توجه و تمرکز بر منابع حمایت اجتماعی درک‌شده در بیماران مبتلا به دیابت نوع 2 با تمرکز بر متغیرهای اجتماعی- اقتصادی، بیش از پیش آشکار می‌شود.

Title: بررسی مقایسه‌ای کیفیت پساب خروجی تصفیه‌خانه‌های فاضلاب صاحبقرانیه، اکباتان و جنوب شهر تهران

http://hsr.mui.ac.ir/index.php/jhsr/article/view/795

مقدمه: امروزه فاضلاب به عنوان یکی از مهم‌ترین منابع آلودگی زیست محیطی محسوب می‌شود و یکی از اهداف تصفیه فاضلاب، سالم نگهداشتن محیط زیست است. تخلیه پساب‌های غیر استاندارد و استفاده از آن‌ها در کشاورزی و یا تخلیه به آب‌های سطحی، مخاطرات بهداشتی و زیست محیطی زیادی را به دنبال خواهد داشت. از این‌رو، مطالعه حاضر با هدف بررسی مقایسه‌ای کیفیت پساب خروجی سه تصفیه خانه تهران (صاحبقرانیه، اکباتان و جنوب) انجام شد. روش‌ها: این مطالعه در تابستان سال 1389 به روش بررسی مقطعی بر روی تصفیه‌خانه‌های پیش‌گفت انجام گرفت. از هر تصفیه‌خانه سه بار نمونه‌برداری انجام شد. پارامترهای اندازه‌گیری شده روی پساب عبارت از pH، DO (Dissolved Oxygen)، 5BOD (Biochemical oxygen demand)، COD (Chemical oxygen demand)، کلراید، سولفات، فسفات، نیترات، نیتریت، TSS (Total suspended solids) و کلیفرم مدفوعی بودند که بر اساس روش‌های معرفی شده در استاندارد متد مورد آنالیز قرار گرفتند. برای تجزیه داده‌ها و رسم منحنی‌های استاندارد به ترتیب از نرم‌افزارهای 12SPSS و Excel استفاده شد. یافته‌ها: مقایسه نتایج مطالعه فعلي با استانداردهای فاضلاب خروجی جهت تخلیه به آب‌های سطحی نشان داد که میانگین غلظت نیتریت در تصفیه‌خانه‌های صاحبقرانیه، اکباتان و جنوب به ترتیب با انحراف معیارهای 02/10، 90/5 و 14/0 برابر با 53/17، 33/50 و mg/l 50/35 و بالاتر از مقدار استاندارد بود. تعداد کلیفرم مدفوعی نیز در پساب هر سه تصفیه‌خانه بیش از استاندارد (ml 100MPN/ 2400) بود. میانگین غلظت پارامتر 5BOD در پساب تصفیه‌خانه جنوب با انحراف معیار 58/4 برابر با mg/l 00/65 بود كه با 5BOD استاندارد اختلاف معنی‌داري نداشت (05/0 < P) و میانگین غلظت COD نیز با انحراف معیار 00/5 برابر با mg/l 00/121 بود که با غلظت COD استاندارد، اختلاف معنی‌داري داشت (05/0 < P) و هر دوی این مقادیر فراتر از حد استاندارد بودند. نتیجه‌گیری: کیفیت پساب تصفیه‌خانه‌های صاحبقرانیه و اکباتان در مقایسه با پساب تصفیه‌خانه جنوب از وضع بهتری برخوردار بود، اما از آن جایی که مقادیر برخی از پارامترها نظیر نیتریت و کلیفرم مدفوعی در این تصفیه‌خانه‌ها بالاتر از حد استاندارد بود، استفاده از پساب این سه تصفیه‌خانه جهت تخلیه به آب‌های سطحی و یا مصارف کشاورزی به دلیل عدم مطابقت با استانداردهای خروجی فاضلاب توصیه نمی‌شود.

Title: بررسی عوامل مؤثر بر دانش کارشناسان مدیریت جهاد کشاورزی شهرستان اصفهان در مورد فن‌آوری نانو

http://hsr.mui.ac.ir/index.php/jhsr/article/view/738

مقدمه: فن‌آوری نانو (Nano technology) به عنوان يک فن‌آوری بين رشته‌اي و پيشتاز رفع مشکلات و کمبود‌ها در بسياري از عرصه‌هاي علمي و صنعتي، به خوبي جايگاه خود را در علوم کشاورزي و صنايع وابسته به اثبات رسانده است. با در نظر گرفتن ضرورت موضوع، مطالعه حاضر با هدف تعیین عوامل مؤثر بر دانش کارشناسان مدیریت جهاد کشاورزی شهرستان اصفهان در مورد فن‌آوری نانو در سال 1391 انجام شد. روش‌ها: مطالعه حاضر از نوع توصیفی تحلیلی به روش مقطعی بود که با سرشماری بر روی کارشناسان شاغل در مرکز مدیریت جهاد کشاورزی شهرستان اصفهان انجام شد. برای جمع‌آوری داده‌ها از پرسش‌نامه محقق ساخته استفاده شد که روایی ظاهری و محتوایی و پایایی پرسش‌نامه مورد تأیید قرار گرفت. اطلاعات به دست آمده با استفاده از ضریب همبستگی Spearman و Pearson و آزمون‌های Mann-whitney U و Kruskal–wallis و نیز تحلیل رگرسیون چند گانه (Multiple regression) به وسیله نرم‌افزار 19SPSS مورد تجزیه و تحلیل قرار گرفت. یافته‌ها: میانگین سنی کارشناسان 5/36 سال با انحراف معیار 4/9 بود. بین متغیر شرکت در دوره‌های آموزشی در ارتباط با فن‌آوری نانو و دانش کارشناسان، ارتباط معنی‌داری وجود داشت (387/0- = r، 000/0 > P)، اما بین سایر متغیرهای مستقل با دانش کارشناسان رابطه‌ی معنی‌داری به دست نیامد. در بررسی عوامل مؤثر بر دانش فن‌آوری نانو، از طريق تحليل رگرسیون، مقدار ضریب تعیین تعديل شده (Adjusted R square) 039/0 به دست آمد. نتیجه‌گیری: دانش کارشناسان كشاورزي نسبت به فن‌آوری نانو در سطح چندان مطلوبی که انتظار می‌رفت، نبود و افرادی که از طریق تحصیلات دانشگاهی با این فن‌آوری آشنا شده بودند، دانش بالاتری داشتند. برای هموار نمودن راه توسعه این فن‌آوری در بخش کشاورزی، اعمال مداخلات جهت ارتقای دانش کارشناسان ضروری به نظر می‌رسد.
